# CRISPR-mediated genome editing allows for efficient on demand creation of >200 kb deficiencies with precise boundaries.

**DOI:** 10.17912/micropub.biology.000949

**Published:** 2023-10-27

**Authors:** Meera Trivedi, Lamine J. Camara, Hannes E. Bülow, Leo T. H. Tang

**Affiliations:** 1 Dominick P. Purpura Department of Neuroscience, Albert Einstein College of Medicine, Bronx, New York, United States; 2 Department of Genetics, Albert Einstein College of Medicine, Bronx, New York, United States

## Abstract

Deficiency mapping remains a useful tool in the process of identifying causative genetic lesions in *C. elegans* mutant strains isolated from forward genetic screens, in particular of non-coding mutants. However, there are significant areas across the genome with no deficiency coverage at all, and the boundaries of many deficiencies remain poorly defined. Here, we describe a simple methodology to generate balanced deficiency strains with up to 230 kb molecularly defined deletions (mini-deficiencies) using CRISPR/Cas9, thus providing a simple path for both precise and tailored deficiency mapping.

**Figure 1. Methods and results on generating mini-deficiencies:**
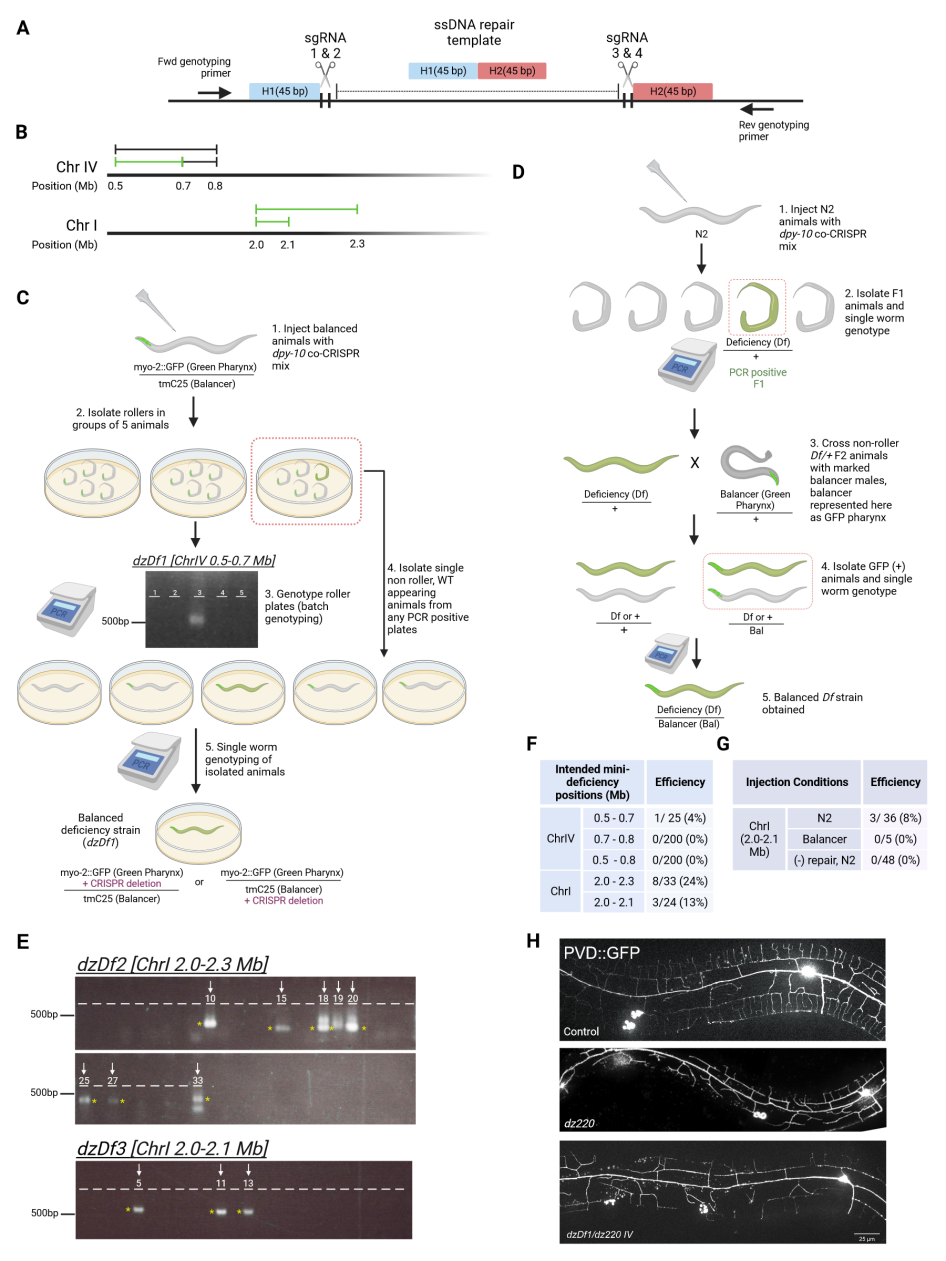
A. Schematic showing the design for gRNA, ssDNA oligo repair, and genotyping primers for generating mini-deficiencies. H1 and H2 denote homology arms. B. Schematic showing the intended (black) and obtained (green) mini-deficiencies. C. Workflow for creating mini-deficiencies by injecting into balanced strains, and batch genotyping to detect successful edits. Agarose gel showing batch genotype result for *dzDf1* . Numbers on gel indicate lane/plate number. D. Workflow for creating mini-deficiencies by injecting into non-balanced strains, and a subsequent cross into balancer males to create a balanced strain. E. Agarose gel showing F1 single worm genotyping results of *dzDf2* and *dzDf3* . Numbers above lanes indicate plate number with arrows marking lanes with PCR bands. Asterisks mark positive PCR results. F. Table summarizing the efficiency in generating the mini-deficiencies. G. Table summarizing efficiency of generating ChrI 2.0-2.1 Mb mini-deficiency under described conditions. H. Images of PVD neuron in wildtype, *dz220* and *dzDf1 / dz220* background, showing non-complementation of *dzDf1* with *dz220* . Created with BioRender.com (https://www.biorender.com/)

## Description

Deficiency mapping remains a key tool in the process of identifying causative genetic lesions in *C. elegans* strains isolated from forward genetic screens. It is a classical mapping technique that relies on deficiency strains, which contain large deletions of the genome from 1-10 Mbs (Fay 2006) . In deficiency mapping, complementation tests between mutants and deficiency strains can rapidly determine whether a mutation of interest lies within or outside of the deleted genomic region in the deficiency (Fay 2006) . This technique is often used to supplement more contemporary mapping approaches involving next-generation sequencing (NGS), which can efficiently identify a region of interest within the genome associated with a specific mutation (Doitsidou et al 2016, Doitsidou et al 2010, Joseph et al 2018, Minevich et al 2012, Smith et al 2016, Zuryn & Jarriault 2013) . Once such a region has been identified through NGS methods, deficiency mapping serves as a simple and rapid way to further pinpoint the causative genetic lesion. It is especially useful when NGS methods identify many mutations within a region of interest or when candidate mutations reside in non-protein coding regions of the genome.

However, deficiency mapping has been greatly limited by two major obstacles: First, to date, deficiencies have been created through random mutagenesis. As a result, the distribution of deficiency strains varies across the genome, with significant areas having no deficiency coverage at all. Second, deficiencies are rarely characterized about their precise boundaries. Third, random deficiencies may contain additional rearrangements that result in non-intuitive complementation patterns with known genes regarding their presumed boundaries, further compromising the practical usefulness of these reagents. To address these limitations, we sought to generate deficiencies in a more precise and controlled manner. To this end, we designed a methodology using CRISPR/Cas9 genome editing, which offers high specificity and flexibility to create large, targeted genomic deletions with defined molecular properties.

Our methodology is in principle not different from routine CRISPR/Cas9 mediated genomic deletion (Dickinson & Goldstein 2016) and is conceptually similar to other CRISPR approaches used to generate balancers and translocations in *C. elegans* (Chen et al 2015, Dejima et al 2018) . However, while past CRISPR/Cas9 approaches have generated deletions up to 2 kb, larger deletions were thought to be too inefficient to generate (Dickinson and Goldstein 2016) . We demonstrate here that simple modifications in the screening of F1/2 progeny allow for the efficient generation of deletions of up to 230 kb. First, two gRNA sites were chosen on each flanking region of the desired deletion ( Figure 1A ). We specifically chose gRNA sites that are predicted to have high on-site efficiency. We then designed a 90nt ssDNA donor repair template based on the gRNA site, consisting of 45nt homologous sequences before the first and after the last gRNA cleavage sites. We designed genotyping primers that flank the repair homology arms. The genotyping PCR should only create a product if the deletion is successful, as the size of the PCR product would be too large for successful amplification from a wild-type chromosome. To account for the fact that the deficiency may encompass recessive lethal genes, we devised two strategies to obtain viable strains: 1) injection into strains containing an appropriate balancer ( Figure 1B ), or 2) immediate crossing with a balanced strain after a deficiency has been created ( Figure 1C ). In all cases, we also employed a *dpy-10* co-conversion strategy to increase our chance of selecting successful edits (Arribere et al 2014) . Below are examples of these two strategies we employed in creating targeted deficiencies.

We employed the first strategy in creating mini-deficiencies on Chr IV, aiming for deletions from ~ 0.5 – 0.8 Mb, 0.5 – 0.7 Mb, and 0.7 – 0.8 Mb ( Figure 1B ). We injected the genome editing mix into the strain RG3191 [ *lem-4 ( ve691 [LoxP + myo-2 p::GFP:: unc-54 3' UTR + rps-27p::neoR:: unc-5 4 3' UTR + LoxP])/tmC25* ]. We chose this strain as it contains a balancer appropriate for our region of interest as well as a GFP marker/recessive lethal allele on the opposite chromosome for easy tracking. To increase the chance of finding successful edits and to reduce manual labor, we performed batch genotyping of the F1s. We first picked 5-10 F1 *dpy-10 /+* rollers onto each plate. We allowed the F1s to self-fertilize and performed whole plate genotyping after the plates were full of animals ( Figure 1C ). We reasoned that if any F1s were successfully edited, our genotyping would be sensitive enough to detect the deletion within the mixed population. In this way, we could effectively increase the number of F1s being screened without increasing the labor involved. After a positive result was obtained, we isolated 20-40 individual wildtype-behaving animals from the PCR positive plate and performed single worm genotyping to identify individual animals with the CRISPR edit. The strain obtained here was already balanced and did not require further hands-on maintenance ( Figure 1C ). One caveat of obtaining mini-deficiencies through CRISPR directly on balancer strains is that the edit can occur on the balancer or the opposing chromosome. Here, the GFP marker allows us to differentiate these two scenarios. We simply crossed the mini-deficiency strain with wildtype males and then genotyped the GFP positive and negative F1s. This step simultaneously generated mini-deficiency males for follow-up complementation tests.

Using this strategy, we were able to successfully create a mini-deficiency deleting 0.5-0.7 Mb of Chr IV. After we pooled 5 F1s per plate to a total of 25 animals, we obtained 1 PCR-positive plate after they had laid a brood ( Figure 1C ). We then isolated 20 individual F2 animals and obtained 6 positive animals. Assuming all these animals were from the same F1, the efficiency of generating this mini-deficiency was 4%. After crossing the deficiency ( *dzDf1* ) into wildtype males, we determined that *dzDf1* resided on the GFP-marked chromosome. However, our effort in creating mini-deficiency deleting 0.5 – 0.8 Mb and 0.7 – 0.8 Mb yielded no edits, despite screening over 200 F1s. We summarized these results in Figure 1F .

While we cannot rule out that our genotyping primers may not be optimized for detecting deletions or the sgRNAs are of low effieicncy, it is also possible that the failure in generating mini-deficiencies may be caused by performing genomic editing on balancer strains. As only half of the progeny from balanced strains are viable and often exhibit phenotypes detrimental to fertility and health, the number and viability of edited F1s may therefore decrease substantially. We therefore devised a second method, where we performed genome editing in an unbalanced background, and then crossed the obtained heterozygous mini-deficiency with a balancer, as illustrated in Figure 1D . To minimize the chance of losing a recessive lethal mini-deficiency, single worm genotyping on the F1s from the injection was performed after they had laid a brood instead of batch genotyping. Once the CRISPR-edited animal was identified, males carrying the marked balancer were crossed with the progenies of the heterozygous mini-deficiency strain. Since these animals are descended from animals heterozygous for the mini-deficiency, genotyping is required on the F1 cross-progeny after they have laid a brood to isolate the balanced deficiency. The positive animal would be the founder of the desired mini-deficiency strain. While this strategy may increase the efficiency of generating mini-deficiencies, it requires more hands-on time in tracking the deficiency before it is properly balanced.

We successfully generated two mini-deficiency in this manner, 2.0 – 2.3 Mb and 2.0 – 2.1 Mb on Chr I ( Figure 1B ). Following injection of the genome editing mix, we genotyped single-worm roller F1s and obtained candidate animals, with genotyping results shown in Figure 1E . In this instance, we obtained 8/33 (24% efficiency) and 3/24 (13% efficiency) of the desired edits for the 2.0 – 2.3 Mbs ( *dzDf2* ) and 2.0 – 2.1 Mb ( *dzDf3* ) deficiencies, respectively ( Figure 1E, F ). We used CGC28 *(+/ szT1 [ lon-2 ( e678 ) umnIs17 ] I; dpy-8 ( e1321 ) unc-3 ( e151 )/ szT1 X)* as our balancer strain, as *szT1* is a well-behaved balancer for the left arm of Chr I and has a high incidence of males. The *umnIs17 [ myo-2 ::GFP]* marker allows for easy identification of cross progeny carrying the balancer. After crossing the balancer males with F2s of the mini-deficiency candidate, we single-worm genotyped the GFP-positive cross progenies F1s and successfully identified mini-deficiency carrying animals. With this methodology, we were able to generate balanced mini-deficiency strains in two weeks. We summarize this result in Figure 1F .

Next, to more directly compare the two methods for generating mini-deficiencies, we injected the CRISPR mix for the ChrI 2.0-2.1 Mb deletion into 10 wild type and 10 CGC28 (balancer) animals. We found that injection into wild type animals more efficiently generated *dpy-10 /+* roller F1s (n=36) as compared to injection into the balanced strain (n=5) despite identical injection mixes ( Figure 1G ). Single-worm genotyping of roller F1 animals revealed an editing efficiency of 3/36 (8%) in the wild type injection versus 0/5 (0%) in the balancer injection ( Figure 1G ). Therefore, we suggest that injection directly into balanced strain may reduce the overall success of obtaining desired edits.

Finally, we utilized the generated mini-deficiency *dzDf1* (Chr IV 0.5 – 0.7 Mb deleted) in mapping *dz220 ,* an allele isolated from a forward genetic screen, that causes defects in the morphology of the multi-dendritic mechanosensory neuron PVD (Diaz-Balzac et al 2016) ( Figure 1H ). Through whole genome sequencing and Hawaiian allele frequency mapping (Doitsidou et al 2010) , we localized the causative mutation in *dz220* animals to a 2 Mb interval between 0-2 Mb of Chr IV. This region contained >20 mutations with no variations resulting in predicted protein changes. A complementation test showed that *dz220* failed to complement *dzDf1* , indicating that the *dz220* mutation is within 0.5 – 0.7 Mb of Chr I. Thus we have successfully narrowed the candidate range from 2 Mb to 0.2 Mb, restricting the number of causative candidate mutations to 3. Follow-up experiments identified the lesion to be at a promoter region. We therefore conclude that mini-deficiencies can greatly aid in mapping lesions found by forward genetics screen as a supplement to whole genome sequencing methods.

While CRISPR/Cas9 genome editing is now routinely performed in *C. elegans* , the feasibility of large edits previously remained unexplored. It was commonly believed that while deletion of a few thousand nucleotides is possible, the efficiency decreases as the size of potential deletions increases (Dickinson & Goldstein 2016) . Here, we demonstrate that a >20% efficiency can be achieved with a 233 kb deletion, suggesting that the upper limit in size of deletion is much higher than previously thought. We also see a high variation of efficiency between different deletions that do not strictly correlate negatively with deletion size. For example, we were able to obtain a 200 kb deficiency for 0.5-0.7Mb on Chr IV but failed to obtain a 100 kb deficiency for 0.7-0. 8Mb ( Figure 1E ). We also see that the efficiency for a 230 kb deletion (24% efficiency for 2.0-2.3 Mb on Chr I) is higher than that for a 100 kb deletion (13% efficiency for 2.0-2.1 Mb, Figure 1E ). This would suggest that the target sites, gRNA, and repair template choice have as much impact on efficiency as the size of the deletion.

DNA repair for double-strand breaks can either be homology-directed repair (HDR) or non-homologous end joining (NHEJ) (Dickinson & Goldstein 2016) . Through direct sequencing of PCR products amplified from our mini-deficiency strains, we observed that each deficiency aligns perfectly with its repair sequence. This indicates that HDR may be the main mechanism for generating these deletions. To further test this hypothesis, we injected the CRISPR mix containing the appropriate sgRNAs but lacking the ssDNA repair template for the ChrI 2.0-2.1 Mb deletion into N2 animals. We isolated and single-worm genotyped 48 *dpy-10 /+* roller F1s. No rollers were PCR-positive for the deletion ( Figure 1G ). In contrast, injection with mixes that included the ssDNA repair template had efficiencies between 8-13% ( Figure 1F, G ). We conclude that the ssDNA repair template is essential or at least beneficial for deficiency generation, suggesting that edits are not primarily generated through NHEJ.

Mini-deficiencies have several advantages over conventional deficiencies for the use of mapping. First, mini-deficiencies can be generated on demand. Since conventional deficiencies are randomly generated, a significant part of the *C. elegans* genome is not covered by any conventional deficiency strain. Our methodology therefore enables deficiency mapping where there is no conventional deficiency coverage. Second, we know the precise boundaries of our CRISPR-generated mini-deficiencies. Conventional deficiencies are seldom sequenced and instead rely on genetic markers to demarcate their boundaries. The interpretation of deficiency mapping can therefore be erroneous due to the ambiguity of the boundary, the possibility of errors in scoring genetic markers, and possible undetectable genome rearrangements. In contrast, mini-deficiencies created through precise genome editing eliminate these sources of uncertainty. Third, we can control the size and location of the mini-deficiencies. Therefore, we can generate multiple overlapping mini-deficiencies to map uncloned genes very effectively. This, in contrast, can only be done with conventional deficiencies if the region of interest is already covered by multiple deficiencies.

## Methods

***C. elegans* maintenance**

All *C. elegans* strains were grown on King’s agar medium plates with *E. coli* ( OP50 ) as a food source at 20ºC.

**CRISPR/Cas9 mediated genome editing**

sgRNA was designed using the IDT Cas9 crRNA design tool. sgRNA and repair templates are listed in the table below. Oligos in the form of TAATACGACTCACTATA(gRNA)GTTTTAGAGCTAGAAATAGCAAG were ordered, where (gRNA) is the 20nt of the guide RNA sequence before the PAM motif, optimized for the T7 promoter. These oligos were used in PCR reactions as a forward primer in conjunction with the universal reverse primer 5’-AAAGCACCGACTCGGTG-3’ with the KOD PCR kit to generate sgRNA transcription templates from the pDD162 plasmid (containing the sequence of the tracrRNA, available from Addgene Plasmid #47549). sgRNA was then transcribed from this template using the HiScribe T7 *in-vitro* transcription kit (NEB) and subsequently purified with the Monarch RNA cleanup kit (NEB). The sgRNA was used in an injection mix at 20 ng/µl concentration per sgRNA, with 250 ng/µl of Alt-R Cas9 endonuclease (IDT) and repair template (100 ng/µl of single strand oligo ordered from IDT). Injection and CRISPR efficiency were monitored through a Co-CRISPR strategy with *dpy-10 ( cn64 )* conversions (Dickinson & Goldstein 2016) .

**Genotyping**

Genomic DNA was extracted from *C. elegans* with Extract-N-amp kit. For single worm genotyping, each animal was suspended in a mixture of 0.8µl extraction buffer and 0.2µl tissue prep buffer, and incubated at 55ºC for 10 minutes followed by 95ºC for 3 minutes. Each lysate is then combined with 0.8µl Neutralization Buffer, followed by PCR master mix comprised of Quickload 2xTaq Master Mix (NEB) and appropriate primers, per the manufacturer's instruction. PCR cycling was performed according to the manufacturer's instructions, and PCR products were visualized on a 1% agarose gel with Ethidium Bromide. For whole plate genotyping, more than 16 worms per plate were collected in a mixture of 2µl of extraction buffer and 0.5ul tissue prep buffer. After heat incubation the lysate was combined with 2µl of Neutralization buffer. 1 µl of lysate was then used as a template for PCR reactions.

**Imaging**

The mechanosensory neuron PVD was labeled with mCherry in the transgene *dzIs53* (Diaz-Balzac et al 2016) *.* Imaging was performed on a Plan-Apochromat 63x/1.4 objective using a Zeiss Axioimager.

## Reagents

***C. elegans s* trains**

The mini-deficiency strains generated in this project are being deposited into CGC.

**Table:** 

**Strain**	**Genotype**	**Available From**
N2	*Caenorhabditis* *elegans*	CGC
RG3191	*lem-4 ( ve691 [LoxP + myo-2 p::GFP::unc-54 3' UTR + rps-27p::neoR::unc-54 3' UTR + LoxP])/tmC25 *	CGC
CGC28	* +/ szT1 [ lon-2 ( e678 ) umnIs17 ] I; dpy-8 ( e1321 ) unc-3 ( e151 )/ szT1 X *	CGC
EB2874	*dzIs53 II; dz220 IV *	Upon request
EB4491	*dzDf1 [IV:506328 - 698511 deleted] lem-4 ( ve691 [ myo-2 p::GFP])/tmC25( unc-5 ) *	CGC
EB4499	*dzDf2 [I:2037935 - 2261431 deleted]/ szT1 [ lon-2 ( e678 ) umnIs17 ] I; szT1 /+ X *	CGC
EB4500	*dzDf3 [I:1999831 - 2100118 deleted]/ szT1 [ lon-2 ( e678 ) umnIs17 ] I; szT1 /+ X *	CGC

**Plasmids**

**Table:** 

**Plasmid**	**Genotype**	**Description**
***pDD162***	*Peft-3::Cas9* + Empty sgRNA	Source for tracrRNA sequence. Addgene Plasmid #47549

**Oligos**

All oligos were ordered from Integrated DNA technologies (IDT)

**Table:** 

**Chr I Deletions**			
gRNAs	At 0.5 Mb	TAATACGACTCACTATAGGCAAAGTCGACCTTGAACTGTTTTAGAGCTAGAAATAGCAAG	
		TAATACGACTCACTATAGTTCTCTATCAAGTATGAGAGTTTTAGAGCTAGAAATAGCAAG	
	At 0.7 Mb	TAATACGACTCACTATAgAGGGGAAACTCCAATCATCGTTTTAGAGCTAGAAATAGCAAG	
		TAATACGACTCACTATAGCTGATAGAAAGCACTACACGTTTTAGAGCTAGAAATAGCAAG	
	At 0.8 Mb	TAATACGACTCACTATAGTGTATCTTCCACAAACTCAGTTTTAGAGCTAGAAATAGCAAG	
		TAATACGACTCACTATAGTGGAAGATACAAGTACACCGTTTTAGAGCTAGAAATAGCAAG	
Repair	0.5 – 0.7 Mb	TCTCCCGATCGATCCAGAAAATGTTGGCAAAGTCGACCTTGAACTGTGTAGTGCTTTCTATCAGCAAGTCTCACGGGGCGCGGCCAATTT	
	0.7 – 0.8 Mb	AGCAAACCAAAAAGCGTAATACAAAAAGGGGAAACTCCAATCATCATCTGTCATGTTCTATGAGACAATTGTAGATCCTTTGGTCATTTT	
	0.5 – 0.8 Mb	TCTCCCGATCGATCCAGAAAATGTTGGCAAAGTCGACCTTGAACTATCTGTCATGTTCTATGAGACAATTGTAGATCCTTTGGTCATTTT	
Genotyping	0.5 Mb Fwd	TCAGCGTACAGCTTCACCAC	
	0.7 Mb Rev	CGAACCAAATCGCTCTGACC	
	0.7 Mb Fwd	CTCGGGCGAATTTGTTGTGT	
	0.8 Mb Rev	GGAACCTCCCAAAGGTACGC	

**Table:** 

**Chr IV 2.0-2.1 Mb Deletion**	
gRNA	TAATACGACTCACTATAgGGCGGATCAAGTCAACTATGTTTTAGAGCTAGAAATAGCAAG
	TAATACGACTCACTATAgCATTATCGGATATTCCTGGGTTTTAGAGCTAGAAATAGCAAG
	TAATACGACTCACTATAgTGAACACTCCGGGAGACCAGTTTTAGAGCTAGAAATAGCAAG
	TAATACGACTCACTATAGGGAGACCATGGAAAACAGGGTTTTAGAGCTAGAAATAGCAAG
Repair	AATATTTCGAAAAAAAATAAATAACTACCATAGAATCAAAAACCGGGGTTCTGGCCTTCCTCATAGAATTTTTCGCGCTCCATTGACAAT
Genotyping	TGCTTATTGCGTTGTATTGGTGT
	TCGGCATAAAAATCATTATAAACGAG
**Chr IV 2.0-2.3 Mb Deletion**	
gRNA	TAATACGACTCACTATAGGCTTGGGAGGCTTCTCACTGTTTTAGAGCTAGAAATAGCAAG
	TAATACGACTCACTATAGATCACAAGATTTTCAGCTTGTTTTAGAGCTAGAAATAGCAAG
	TAATACGACTCACTATAGCTACGTAGGAAAGATAGGAGTTTTAGAGCTAGAAATAGCAAG
	TAATACGACTCACTATAGTGGTTGGATTCCACTACGTGTTTTAGAGCTAGAAATAGCAAG
Repair	AGCTTCAGACGCGTTTTCACTGAAAAATGTGAATTTGCTAGGGAGGTCGCAGTATACAAACGTCTGTACAAATCTTCTTTGGCAGAGCCA
Genotyping	TAATGCCCCGATCTACTGCG
	ATACTGCGACCTCCCTAGCA
